# KDEL receptor 1 regulates T-cell homeostasis via PP1 that is a key phosphatase for ISR

**DOI:** 10.1038/ncomms8474

**Published:** 2015-06-17

**Authors:** Daisuke Kamimura, Kokichi Katsunuma, Yasunobu Arima, Toru Atsumi, Jing-jing Jiang, Hidenori Bando, Jie Meng, Lavannya Sabharwal, Andrea Stofkova, Naoki Nishikawa, Hironao Suzuki, Hideki Ogura, Naoko Ueda, Mineko Tsuruoka, Masaya Harada, Junya Kobayashi, Takanori Hasegawa, Hisahiro Yoshida, Haruhiko Koseki, Ikuo Miura, Shigeharu Wakana, Keigo Nishida, Hidemitsu Kitamura, Toshiyuki Fukada, Toshio Hirano, Masaaki Murakami

**Affiliations:** 1 Division of Molecular Neuroimmunology, Institute for Genetic Medicine and Graduate School of Medicine, Hokkaido University, Kita-15, Nishi-7, Kita-ku, Sapporo 060-0815, Japan; 2 Laboratory of Developmental Immunology, Graduate School of Frontier Biosciences, Graduate School of Medicine, and WPI Immunology Frontier Research Center, Osaka University, 2-2, Yamada-oka, Suita 565-0871, Japan; 3 Radiation Biology Center, Kyoto University, Yoshida-Konoe-cho, Sakyo-ku, Kyoto 606-8501, Japan; 4 Laboratory for Developmental Genetics, RIKEN Research Center for Allergy and Immunology, 1-7-22 Suehiro-cho, Tsurumi-ku, Yokohama 230-0045, Japan; 5 Laboratory for Immunogenetics, RIKEN Research Center for Allergy and Immunology, 1-7-22 Suehiro-cho, Tsurumi-ku, Yokohama 230-0045, Japan; 6 Technology and Development Team for Mouse Phenotype Analysis, RIKEN Bioresource Center, 3-1-1 Koyadai, Tsukuba 305-0074, Japan; 7 Laboratory for Cytokine Signaling, RIKEN Research Center for Allergy and Immunology, 1-7-22 Suehiro-cho, Tsurumi-ku, Yokohama 230-0045, Japan; 8 Osaka University, 2-1, Yamada-oka, Suita 565-0871, Japan

## Abstract

KDEL receptors are responsible for retrotransporting endoplasmic reticulum (ER) chaperones from the Golgi complex to the ER. Here we describe a role for KDEL receptor 1 (KDELR1) that involves the regulation of integrated stress responses (ISR) in T cells. Designing and using an *N*-ethyl-*N*-nitrosourea (ENU)-mutant mouse line, T-Red (naïve T-cell reduced), we show that a point mutation in KDELR1 is responsible for the reduction in the number of naïve T cells in this model owing to an increase in ISR. Mechanistic analysis shows that KDELR1 directly regulates protein phosphatase 1 (PP1), a key phosphatase for ISR in naïve T cells. T-Red KDELR1 does not associate with PP1, resulting in reduced phosphatase activity against eIF2α and subsequent expression of stress responsive genes including the proapoptotic factor Bim. These results demonstrate that KDELR1 regulates naïve T-cell homeostasis by controlling ISR.

## Abstract (web summary)

KDEL receptors are known to be involved in retrotransporting chaperones to the endoplasmic reticulum from the Golgi complex. Here the authors unravel a role of KDEL receptor 1 in regulating integrated stress responses in naïve T cells through its association with protein phosphatase 1.

## Graphical abstract

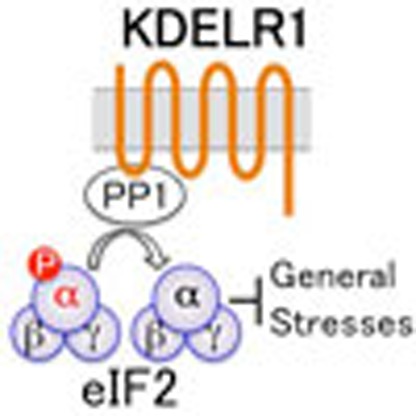

KDEL receptor 1 (KDELR) was originally found to be responsible for the return of soluble endoplasmic reticulum (ER)-resident proteins to the ER from the intermediate compartment of the *cis*-Golgi^1^,^2^. This retrograde transport requires soluble ER-resident proteins to either have a KDEL-like motif at their C terminus or to form a complex with ER-resident proteins that do^3^,^4^. Consistently, it has been reported that KDELR modulates ER stress responses, at least in HeLa cells^5^. A more recent study has suggested that KDELR function goes beyond motif recognition by demonstrating that the chaperone-bound KDELR triggers the activation of Src family kinases at the Golgi complex, a phenomenon that may be critical for intracellular signalling cascades^6^,^7^.

Integrated stress responses (ISR) are general stress-response programmes conserved from yeast and known to modulate cellular homeostasis by integrating various types of stress signals, including ER stress, amino-acid deprivation, infection with double-stranded RNA viruses, haem deficiency and oxidative stress^8^,^9^,^10^. These diverse signals increase the activation status of four stress kinases—double-stranded RNA-dependent protein kinase R (PKR), RNA-dependent protein kinase-like ER kinase (PERK), eukaryotic initiation factor 2 (eIF2α) kinase general control non-repressed 2 (GCN2) and haem-regulated eIF2α kinase, each of which regulates phosphorylation at serine 51 of the α subunit of eIF2. This eIF2α modification generally attenuates translation, while the activation of ISR via eIF2α alteration mobilizes the expression of stress-induced genes involved in apoptosis induction, including Bim, CHOP and Trib3 (ref. 11). Furthermore, prolonged phosphorylation of eIF2α induces apoptosis^12^,^13^. In particular, protein phosphatase 1 (PP1) interacts with the regulatory proteins GADD34 and CreP to reduce eIF2α phosphorylation^14^. In addition, defects in ISR are associated with the development of several important pathologies, including diabetes, Alzheimer's disease and viral infection^15^,^16^,^17^. Although ISR can affect the differentiation and activation status of T cells^18^,^19^, it remains unknown whether they also play a role in the homeostasis of naïve T cells in steady state *in vivo*.

The number of T cells is relatively constant in the body. When peripheral T cells are reduced due to thymic involution due to aging, infections or irradiation, the remaining T cells proliferate to act as a compensatory mechanism for homeostatic proliferation, a response induced by TCR signalling and cytokines^20^. The apoptosis of peripheral T cells is mainly regulated by Bim^21^,^22^,^23^,^24^, and Bim expression is controlled by Bim transcriptional levels and under stress conditions induced by the transcription factor Chop rather than the forkhead box O family^25^.

We here establish a new ENU-induced mutant mouse line that shows a decreased number of naïve T-cell numbers, which we name T-Red (naïve T-cell reduced). T-Red mice have a point mutation in KDELR1, and dysfunctional KDELR1 is responsible for the reduction of naïve T cells. T-cell-mediated responses are attenuated in T-Red mice, and mechanistic analysis suggests that KDELR1 regulates ISR in naïve T cells via PP1 activity. Phosphorylation of eIF2α is enhanced due to a reduction in activity of PP1 in T-Red naïve T cells, and T-Red KDELR1 does not associate with PP1. Indeed, naïve T cells in T-Red mice increase the targets of the eIF2α pathway, such as proapoptotic factors like Bim, CHOP and Trib3, to eventually cause apoptosis. Thus, we suggest that KDELR1 regulates ISR by controlling PP1 activity in naïve T cells *in vivo*.

## Results

### A mutant strain with excess memory/activated T cells

**Figure 1:**
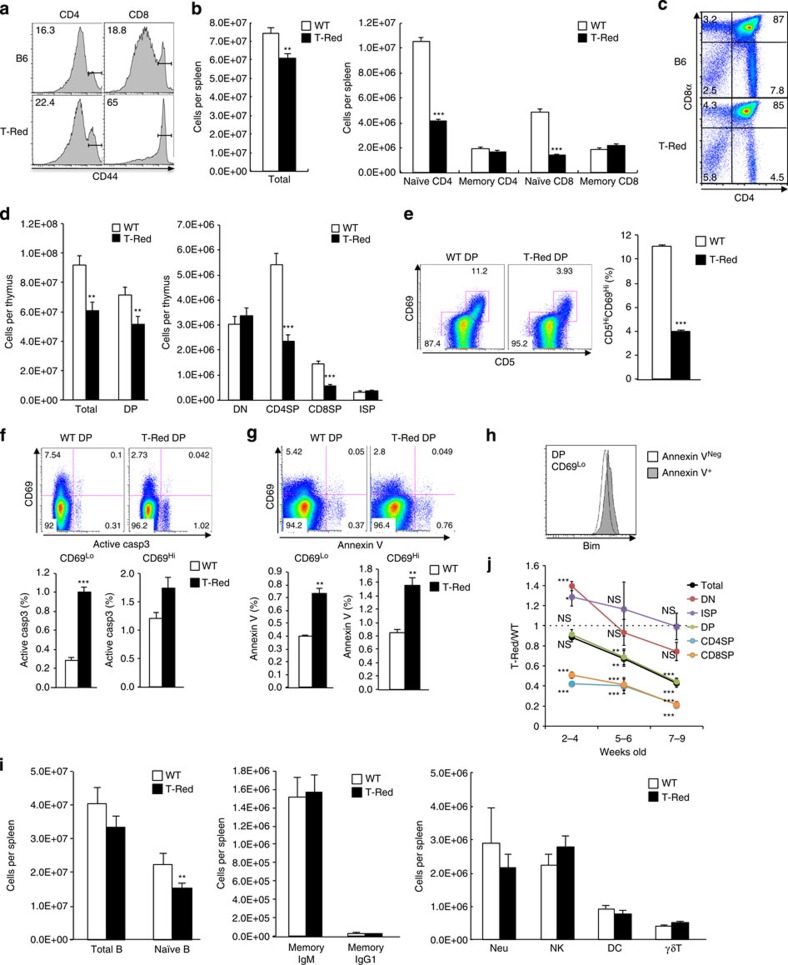
Establishment of a mutant mouse strain having excess memory T cells. (**a**) T-cell phenotypes in a mutant strain induced by ENU treatment (T-Red) and control (B6) mice. Flow cytometry analysis was performed using peripheral blood from the mutant strain (9 weeks old). Indicated numbers are percentages of memory/activated phenotype T cells, as monitored by CD44 expression. (**b**) Number of T cells harvested from the spleen. Mice up to 12 weeks old were used. (**c**) CD4 and CD8 plots of the thymus at 6 weeks old. (**d**) Number of thymocytes harvested from the thymus. DP, CD4+CD8+ population; DN (double-negative), CD4-CD8- population; CD4SP (CD4 SP), CD3highCD4+CD8- population; CD8SP (CD8 SP), CD3highCD4-CD8+ population; and ISP (immature SP), CD3 lowCD4-CD8+ population. Mice up to 12 weeks old were used. (**e**) CD5 and CD69 levels in CD4+CD8+ DP population (left) and the frequency of CD5^Hi^CD69^Hi^ DP populations (right). Mice at 6–8 weeks old were used. (**f**,**g**) The frequencies of active caspase 3 (casp3) (**f**) and annexin V (**g**) in DP thymocytes. (**h**) Bim levels of annexin V-negative (Neg) or positive (+) DP CD69Lo thymocytes. (**i**) Cell numbers of other cell types in the spleen: B (CD19+) cells, naïve B (CD19+IgM+CD273-) cells, memory IgM (CD19+IgM+CD273+) cells, memory IgG1 (CD19+IgG1+CD273+) cells, natural killer cells (NK), γδT cells (γδT), neutrophils (Neu) and dendritic cells (DC). Mice up to 12 weeks old were used. (**j**) Time course of changes in thymic cellularity. The ratio (T-Red/WT) of each thymic population (cell number) is shown. Data represent the mean+s.e.m. (**b**, *n*>40; **d**, *n*>30; **e**–**g**, *n*=4–6 mice; **i**, *n*=8–20; and **j**, *n*=6–14 for each time point). Representative FACS plots from more than three independent experiments are shown (**c**,**e**–**h**). *P* values are shown or indicated by asterisks (*P<0.05, **P<0.01 and ***P<0.001); NS, not significant.

A mouse library with random genome-wide point mutations was generated by treating C57BL/6 male mice with the chemical mutagen ethylnitrosourea (ENU)^26^. A first-generation male offspring was bred with wild-type (WT) C57BL/6 female mice, and the second generations were intercrossed. In total, 309 offspring from the third-generation pedigrees were screened for T-cell phenotypes in the peripheral blood by a flow cytometer to identify mutants with aberrant T-cell homeostasis *in vivo*. In one pedigree, several mice exhibited an unusually high percentage of CD44 expression on the cell surface, which represents the memory/activated T-cell phenotype (Fig. 1a). This phenotype was inherited as a simple autosomal recessive trait (Table 1) and was more evident in CD8+ T cells than in CD4+ T cells (Fig. 1a).

To distinguish whether it was the number of memory/activated T cells that increased or the number of naïve T cells that decreased, we counted T-cell numbers in lymphoid organs. The total cell number in the spleen was reduced in ENU-mutant mice (Fig. 1b). More specifically, the number of CD44lowCD62Lhigh naïve T cells significantly decreased in the spleen (Fig. 1b, Supplementary Fig. 1). Total cell numbers in the thymus, double-positive (DP) and single-positive (SP) thymocytes were also less in mutant mice (Fig. 1c,d). To investigate a specific blockade point in the ENU-mutant thymus, we performed flow cytometry analysis and found a reduction of positive selecting thymocytes (CD4+CD8+CD5lowCD69low) and positive selected thymocytes (CD4+CD8+CD5highCD69high), but an increase of dying thymocytes (Bimhigh, Casp3high or Annexin Vhigh in CD4+CD8+CD5lowCD69low thymocytes), suggesting that thymocyte development was inhibited during the selection process in mutant mice (Fig. 1e–h). On the other hand, other cell populations, including CD44 high memory/activated phenotype T cells, double-negative (DN) and CD8+CD3low immature SP (ISP) thymocytes in the thymus and natural killer cells, γδT cells, neutrophils and dendritic cells of the spleen, were unaffected (Fig. 1b,d,i). We also found that the number of naïve B cells but not memory B cells was reduced (Fig. 1i). Interestingly, the T-cell phenotypes described above were more evident after the weaning period (after 5-6 weeks; Fig. 1j). In addition, phenotype differences between T cells from T-Red mice and those from control mice became more apparent with the developmental stage of T cells, as shown in Fig. 1b,d (DP thymocytes<SP thymocytes<naïve T cells). These results demonstrate that the number of T-cell linage cells significantly decreased in T-Red mice after the DP thymocyte stage, which is when thymocytes obtain TCR complex molecules. We hypothesize that the reduction of thymocytes is induced by their longer lifetime in the thymus due to an accumulation of thymocyte social stress. Thus, we concluded that the T-cell phenotype in the periphery of mutant mice is due to fewer naïve T cells, not more memory/activated T cells. Because memory/activated T cells originate from naïve T cells, it is likely that the near normal number of memory/activated T cells in the mutants was caused by homeostatic proliferation^27^,^28^. We therefore named this mutant strain ‘T-Red' (naïve T-cell reduced).

### A point mutation in *Kdelr1* is responsible for the phenotype

**Figure 2:**
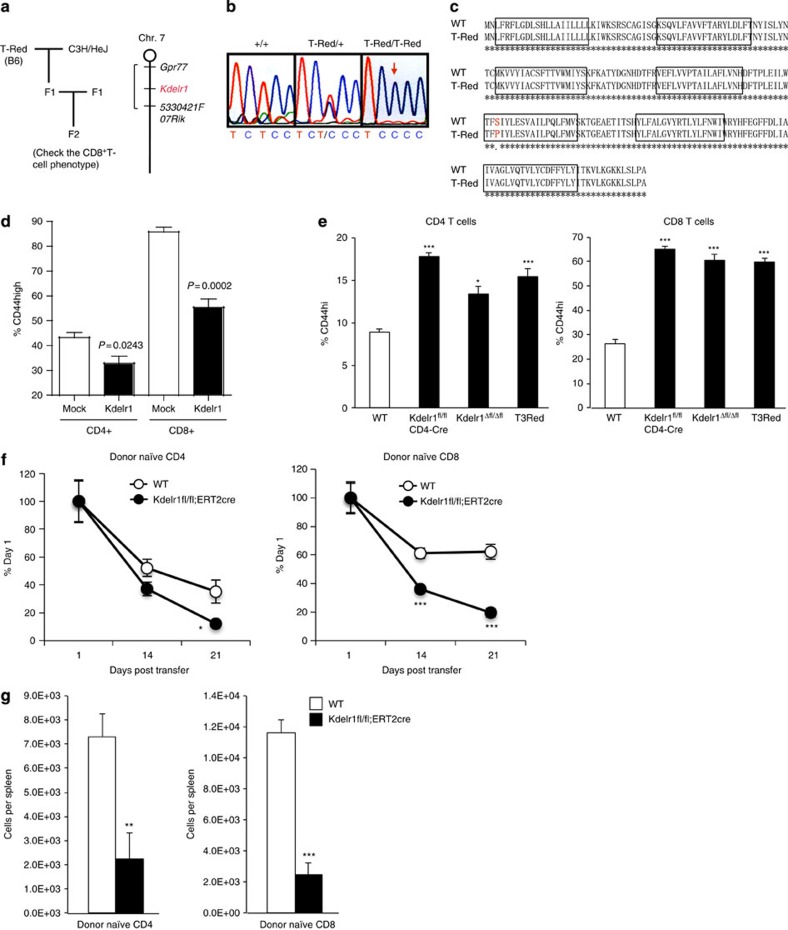
A point mutation in the *Kdelr1* gene is responsible for the T-Red T-cell phenotype. (**a**) Scheme of the gene mapping (left). The chromosomal location of the gene responsible for T-Red was mapped in an ∼100 kb region between the Gpr77 and 5330421F07Rik genes of chromosome 7 (right). (**b**) Resequencing mRNA and genomic DNA exons revealed a single T→C nucleotide substitution in the *Kdelr1* gene mouse homologue. (**c**) This mutation resulted in a Ser123→Pro amino-acid substitution in the fifth transmembrane domain. Boxes represent presumptive transmembrane domains. (**d**) Flow cytometry analysis of CD4+ and CD8+ T-cell phenotypes 2 months after retrovirus-mediated forced expression of WT KDELR1 (Kdelr1) or control vector (mock) in T-Red mouse derived haematopoietic stem cells transplanted into the bone marrow (5–6 weeks old). Percentages of CD44 high populations of T cells are shown. Data represent the mean+s.e.m. (*n*=3–4). (**e**) Flow cytometery analysis in T cells was performed using peripheral blood from T-Red, CD4-cre/ *Kdelr1*^flox/flox^, CAG-Cre/ *Kdelr1*^flox/flox^ (*Kdelr1*^Δfl/Δfl^) and control mice (7–14 weeks old). Percentages of CD44 high populations of T cells in peripheral blood are shown. Data represent the mean+s.e.m. (*n*=6–23). One-way ANOVA with *post hoc* Dunnett's test was used. (**f**) The percentages in peripheral blood (% of day 1) of donor naïve CD4 or CD8 T cells from WT and *Kdelr1*^flox/flox^;ERT2cre (8–11 weeks old) mice. The cells were mixed 1:1 on transfer. The host mice were treated with tamoxyfen to induce Kdelr1 deficiency after transfer. (**g**) The absolute numbers of donor cells in the spleen of the mice in **f** on day 21. Data represent the mean+s.e.m. (*n*=6). *P* values are shown or indicated by asterisks (*P<0.05, **P<0.01 and ***P<0.001).

By using F2 mice intercrossed between C57BL/6T-Red homozygotes and a WT C3H/He strain, the chromosomal location of the genes responsible for the T-cell phenotype in T-Red mice was mapped within an ∼100-kb region of chromosome 7, which contains 443 genes (Fig. 2a). Resequencing the mRNA and genomic DNA exons of T-Red mutants within this region revealed a single T→C nucleotide substitution in a gene identified as the mouse homologue of *Kdelr1* (Fig. 2b). This mutation resulted in a Ser123→Pro amino-acid substitution within the fifth transmembrane region of *Kdelr1* (Fig. 2c).

To prove whether this point mutation is responsible for the T-cell phenotype in T-Red mice, we performed two experiments—a retrovirus-mediated rescue experiment using the WT *Kdelr1* gene and the design and analysis of *Kdelr1* knockout mice. Forced expression of the WT *Kdelr1* gene in T-Red-derived haematopoietic stem cells followed by bone marrow transplantation (BMT) increased the percentage of naïve T cells while concomitantly reducing the memory/activated T-cell fraction, as seen by the decreased surface CD44 expression (Fig. 2d). Furthermore, systemic (*Kdelr1*^Δflox/Δflox^ mice) and T-cell-specific (CD4-Cre/ *Kdelr1*^flox/flox^ mice) deletions of the *Kdelr1* gene resulted in almost the same T-cell phenotype as that of T-Red mice (Fig. 2e).

We also examined whether the T-Red phenotype corresponds to the physiological function of KDELR1 molecules. We performed several detailed experiments on mice having deletions of the *Kdelr1* gene in T cells (*Kdelr1*^flox/flox^ mice crossed with CD4-Cre mice). Data from *Kdelr1*-deficient mice (Supplementary Fig. 2) were very similar to the data from T-Red mice. In addition, we transferred naïve T cells from *Kdelr1*^flox/flox^-ERT2-Cre mice into WT hosts and deleted *Kdelr1* by treatment with tamoxyfen. Both naïve CD4+ T cells and CD8+ T cells were reduced after the tamoxyfen administration (Fig. 2f,g). Therefore, we concluded that the T-Red phenotype corresponds to the physiological function of KDELR1 molecules, at least in T cells, and that the T-Red mutation in the *Kdelr1* gene is responsible for the T-Red T-cell phenotype and the loss of function of KDELR1 molecules.

### T-cell responses are attenuated in T-Red mice

**Figure 3:**
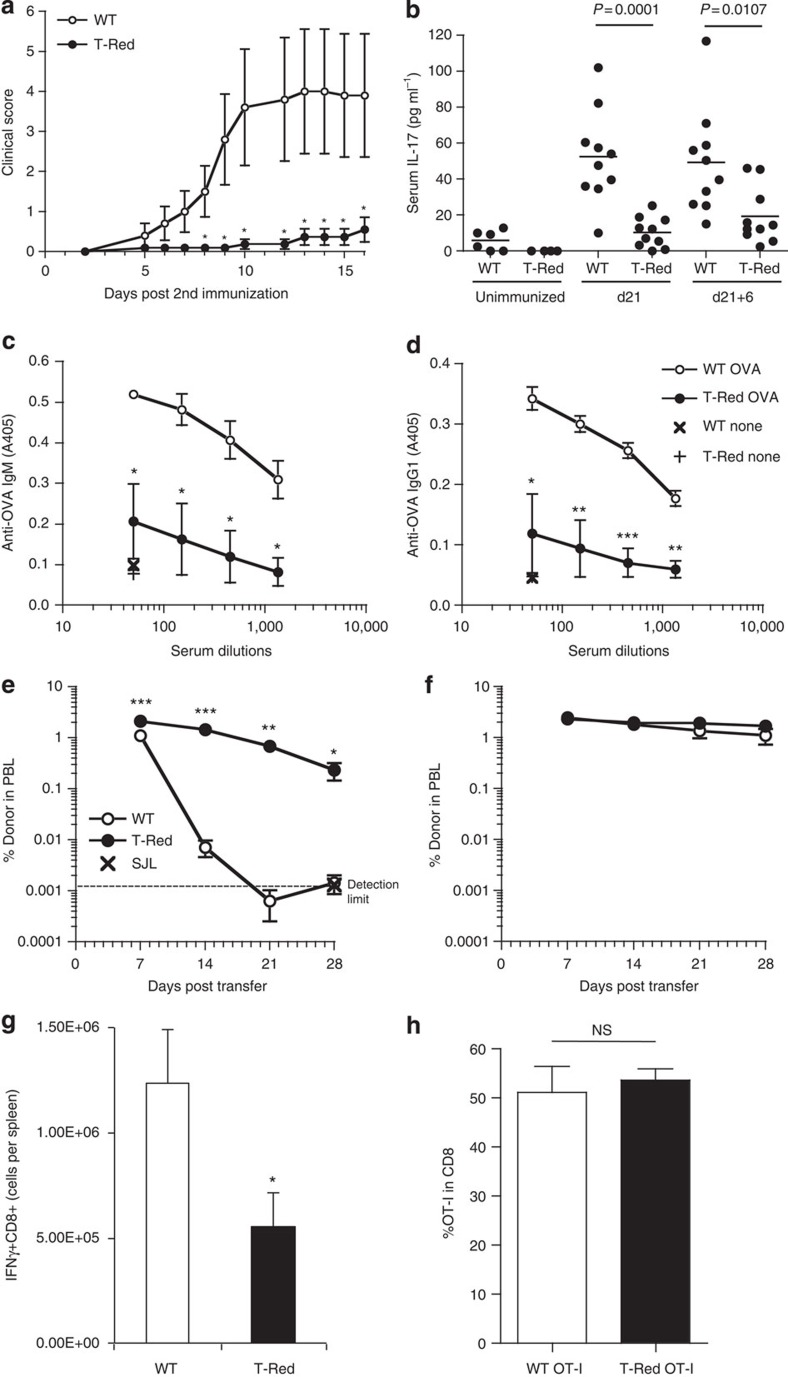
Antigen-specific T-cell responses were attenuated in T-Red mice. (**a**,**b**) Collagen-induced arthritis model. Clinical scores (**a**) and serum concentrations of IL-17 (**b**) in T-Red and control mice (5–8 weeks old). Serum IL-17A concentrations were measured by ELISA before antigen immunization (unimmunized), 21 days after immunization (d21) and 6 days after secondary immunization on day 21 (d21+6). (**c**,**d**) T-cell dependent response to OVA. Serum concentrations of the anti-OVA IgM (**c**) and anti-OVA IgG1 (**d**) were measured in T-Red and control mice (7–9 weeks old) after immunization with OVA in the presence of alum. (**e**,**f**) Male cell rejection in female mice. Congenic CD45.1 male (**e**) or female (**f**) splenocytes were transferred into female T-Red or control mice (6–8 weeks old). Percentages of the transferred donor cells in peripheral blood (PBL) are shown. (**g**,**h**) Bacteria infection. *Listeria monocytogenes* expressing OVA was infected in T-Red and control mice (6–8 weeks old). Cell numbers of OVA-specific IFNγ+ populations in CD8+ T cells on day 7 post infection are shown (**g**). The same number (20,000 cells) of OT-I or T-Red/OT-I cells was transferred into WT congenic hosts 1 day before infection. The frequency of donor cells in the blood CD8+ T cells was determined on day 7 post infection (**h**). Data represent the mean±s.e.m. (**a**, *n*=8–10; **c**–**f**, *n*=4; and **g**–**h**, *n*=4–5). Wilcoxon test was used in **a**. *P<0.05; **P<0.01; ***P<0.001. NS, not significant.

To investigate whether the reduced number of naïve T cells in T-Red mice has any impact on antigen-specific T-cell responses, we employed four experimental systems *in vivo*. In collagen-induced arthritis, clinical scores and serum concentrations of interleukin (IL)-17A were significantly decreased in T-Red mice (Fig. 3a,b). Serum concentrations of anti-ovalbumin (OVA) antibodies after immunization with OVA/Alum were also significantly inhibited in the mutant (Fig. 3c,d). In addition, male, but not female, antigen-specific rejection in female mice was attenuated (Fig. 3e,f), and the CD8+ T-cell response against *Listeria monocytogenes*-OVA was less in T-Red mice (Fig. 3g). However, when the same numbers of naïve OT-I and T-Red/OT-I CD8+ T cells, which have OVA-specific TCR due to a rearranged TCR transgene, were transferred, the expansion of these cells in response to *L. monocytogenes*-OVA infection was found equivalent (Fig. 3h). Similarly, *in vitro* proliferation and Th17 differentiation were not significantly impaired in T-Red naïve T cells after stimulation with anti-CD3 antibody (Supplementary Fig. 3). We also confirmed that male antigen-specific rejection in female mice was attenuated in mice having T-cell-specific deletions of the *Kdelr1* gene (Supplementary Fig. 2e). Thus, antigen-specific T-cell responses were attenuated in T-Red mice, most likely because of reduced naïve T-cell numbers via the functional defect of KDELR1 molecules. While it is possible that a shorter longevity of animals may occur in certain conventional conditions due to a reduction of T cells, we observed that T-Red mice had normal longevity and no clear abnormalities even with age in the specific pathogen-free conditions.

### Pre-rearranged TCR rescues naïve T-cell reduction

**Figure 4:**
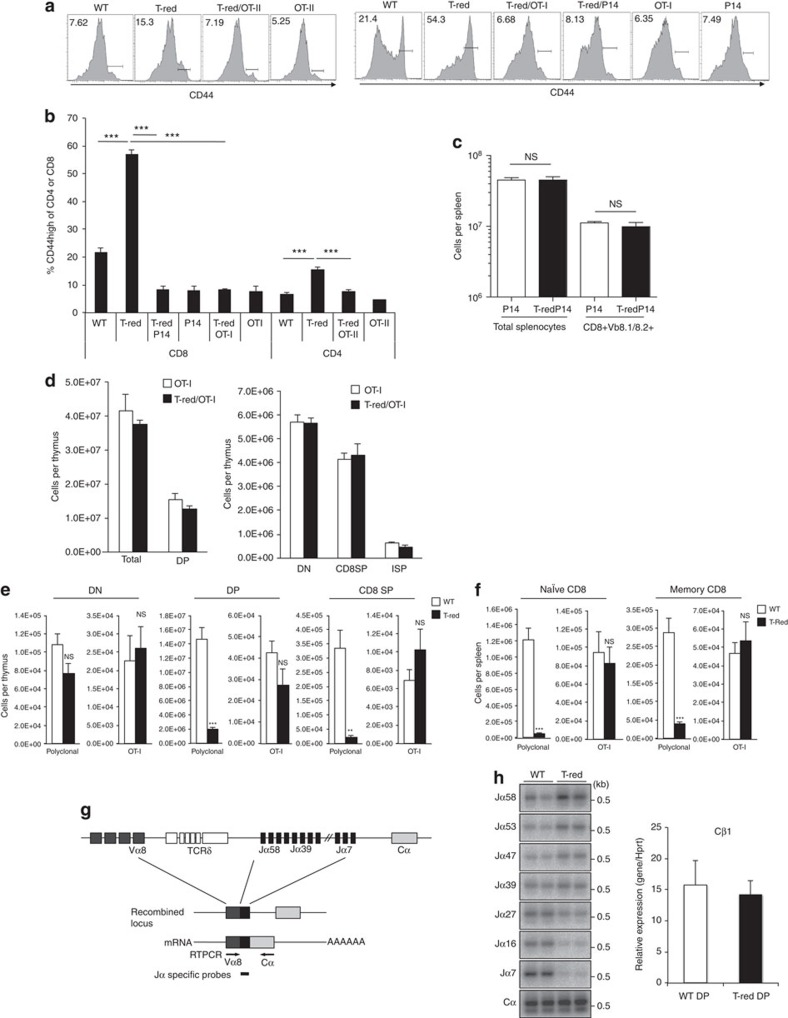
Pre-rearranged TCR corrected the T-Red phenotype. (**a**,**b**) Histograms of CD44 levels (**a**) and percentages of the CD44 high memory/activated T-cell phenotype (**b**) in peripheral blood of P14, OT-I and OT-II TCR transgenic mice of T-Red- or WT backgrounds. Mice up to 12 weeks old were used. Representative FACS plots from more than three independent experiments are shown. One-way ANOVA with *post hoc* Dunnett's test was used in **b**. (**c**) Total number of splenocytes and CD8+Vβ8.1/8.2+ T cells in P14 TCR transgenic mice (Vβ8.1+) of T-Red or WT backgrounds (6–8 weeks old). (**d**) Absolute cell numbers of thymocyte subpopulations in OT-I and T-Red/OT-I mice (8–9 weeks old). (**e**,**f**) Bone marrow cells from WT (CD45.2/CD90.1), T-Red (CD45.1/CD45.2/CD90.2), OT-I (CD45.2/CD90.1/CD90.2) and T-Red/OT-I (CD45.2/CD90.2) mice were mixed and transplanted into lethally irradiated WT mice (CD45.1/CD90.2; 8–10 weeks old). Each population was tracked by a flow cytometer using congenic markers. Absolute cell numbers of the chimera in the thymus (**e**) and spleen (**f**) are shown. (**g**) Schematic representation of the TCR Vα–Jα–Cα boundary. (**h**) DP thymocytes were sorted from T-Red and control mice (8–9 weeks old) and isolated from total RNA. RT-PCR was performed using Vα8- and Cα-specific primers followed by southern blotting with Jα- or Cα-specific probes (left). Representative images from three independent experiments are shown. Cβ1 mRNA levels were examined by quantitative PCR (right). Relative expression levels to Hprt are shown. Data represent the mean+s.e.m. (**b**, *n*=7–39; **c**, *n*=4; **d**, *n*=4–6; **e**,**f**, *n*=9; **h**, *n*=3). Paired Student's *t*-test was used in **f**. **P<0.01 and ***P<0.001. NS, not significant.

We found that CD44 levels of T-Red OT-I T cells were significantly reduced compared with T-Red CD8+ T cells but comparable to WT OT-I T cells (Fig. 4a). Therefore, additional lines of T-Red TCR transgenic strains were generated. Again, the percentages and numbers of naïve T cells did not show any dramatic decrease in P14, OT-I and OT-II TCR transgenic mice under the T-Red background (Fig. 4a–c). We also found that there was a minimum difference between thymic numbers in OT-I transgenic WT and OT-I T-Red mice (Fig. 4d).

We performed BMT experiments using WT and T-Red mice or regular OT-I and T-Red OT-I mice to further explore the link between the pre-rearranged TCR and T-Red phenotype. The BMT experiments showed results similar to those presented above, as we found a smaller T-cell population in T-Red-derived BM cells but not in WT-derived BM cells (regular or OT-I case; Fig. 4e,f). All these results suggest that the reduction of naïve T cells in T-Red mice is dependent on an incomplete TCR rearrangement process and/or TCR signal transduction process in some T-cell repertoires in the thymus and in naïve T cells in the periphery.

### TCR rearrangement in T-Red mice is essentially complete

Because T-Red mice with TCR transgenic backgrounds showed normal percentages of naïve T cells (Fig. 4a–f), we considered whether the functional defect of KDELR1 induces an incomplete TCR rearrangement process to induce the stress that is stimulated by DNA damage responses. Although TCR Jα utilization was perturbed in T-Red T cells, with proximal TCR Jα fragments from TCR Vα being more rearranged than distal ones (Fig. 4g,h), the total amount of rearranged TCR was equivalent according to a Cα probe as well as qPCR of Cβ (Fig. 4h). We also found normal TCRβ rearrangements, which were induced by DNA segments in a narrower region compared with TCRα segments^29^,^30^, in DP thymocytes of T-Red mice and showed normal usage of TCRβ molecules in naïve CD4+ and naïve CD8+ T cells in T-Red mice (Supplementary Fig. 4). These results strongly suggest that TCR rearrangement in T-Red mice was essentially complete. Therefore, we concluded that the reduction of naïve T cells in T-Red mice is not dependent on an incomplete TCR rearrangement process. Instead, the reduction of naïve T cells in T-Red mice may be dependent on a TCR signal transduction process in some T-cell repertoires of the thymus and in naïve T cells of the periphery.

### Bim and apoptosis increased in T-Red naïve T cells

**Figure 5:**
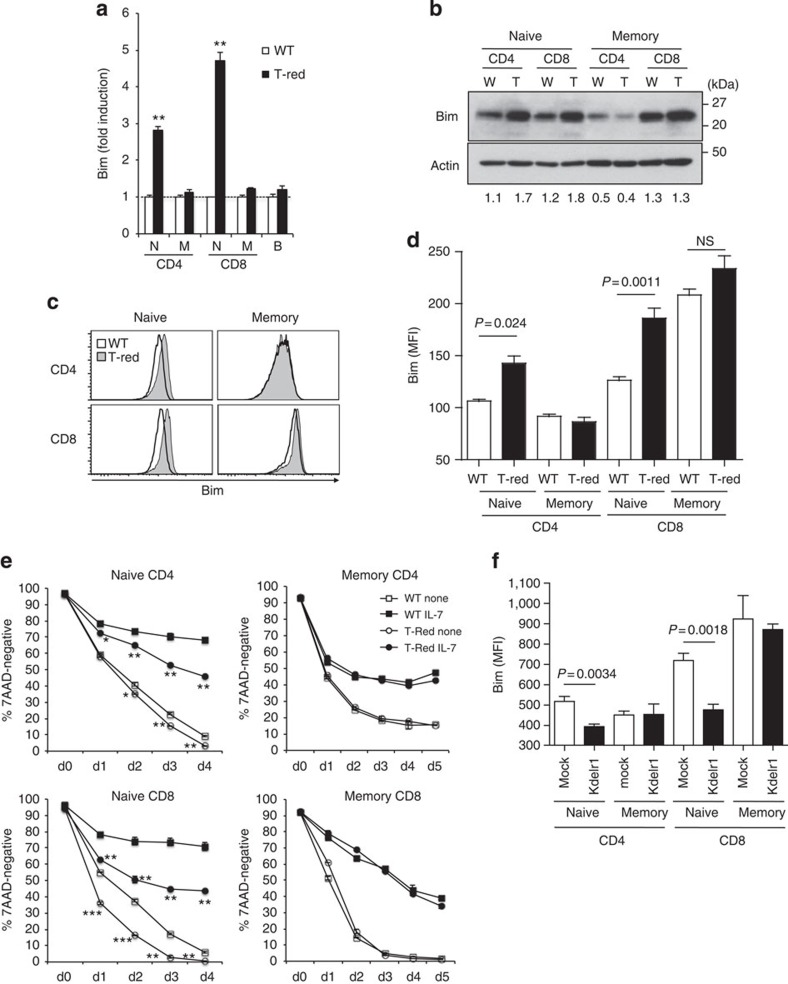
A functional defect in KDELR1 increases stress-mediated Bim expression and apoptosis in T-Red naïve T cells. (**a**–**d**) Bim mRNA and protein levels were investigated by real-time PCR (**a**), western blot (**b**) and flow cytometry (**c**,**d**) in T-Red (T) and WT (W) mice (8–10 weeks old). N, M and B indicate naïve, memory and B cells, respectively. Expression levels of WT populations were normalized as 1 in **a**. Numbers in **b** represent the intensity ratio of Bim/Actin. Representative images from three independent experiments are shown in **b**,**c**. The mean fluorescence intensity (MFI) of intracellular staining of Bim is shown in **d** (*n*=3–4). (**e**) *In vitro* survival of naïve and memory/activated T cells in the presence or absence of IL-7. Mice between 9 and 10 weeks old were used. (**f**) Bim expression in naïve and memory/activated T cells by flow cytometry following retrovirus-mediated forced expression of WT KDELR1 in T-Red haematopoietic stem cells and BMT (*n*=4). Mice between 6 and 8 weeks were used. Data represent the mean+s.d. (**a**,**e**) or s.e.m. (**d**,**f**). *P* values are shown in some figures. *P<0.05; **P<0.01; ***P<0.001. NS, not significant.

We next investigated why the number of naïve T cells in T-Red mice is lower than in control mice. We first considered whether naïve T cells from T-Red mice undergo higher rates of apoptosis and examined the expression levels of the proapoptotic factor Bim, a major apoptosis inducer in T cells, particularly in the periphery^21^,^22^,^23^,^24^. T-Red mice showed significantly higher expressions of Bim in their naïve T cells, but not in their memory/activated T cells or B cells when compared with controls (Fig. 5a–d). Consistent with these differences in Bim quantity between memory/activated CD4+ T cells and CD8+ T cells (Fig. 5b), it is known that memory CD8+ T cells have more and memory CD4+ T cells fewer Bim molecules than naïve T cells^31^ and that memory CD8+ T cells are resistant to increased expression of Bim in a manner dependent on the expression of Bcl-2 (ref. 32). Indeed, T-Red naïve T cells, but not memory/activated ones, showed lower survival rates in the presence or absence of IL-7 (Fig. 5e). We also found that *Kdelr1* deficiency in naïve T cells induced more apoptosis in both CD4+ and CD8+ T cells in the presence or absence of IL-7 (Supplementary Fig. 2d). Furthermore, forced expression of the WT *Kdelr1* gene decreased Bim expression in naïve T-Red T cells, but not in memory/activated ones (Fig. 5f). These results suggest that a functional defect in KDELR1 induces the expression of Bim to cause apoptosis in naïve T cells.

### ISR is enhanced in T-Red naïve T cells

**Figure 6:**
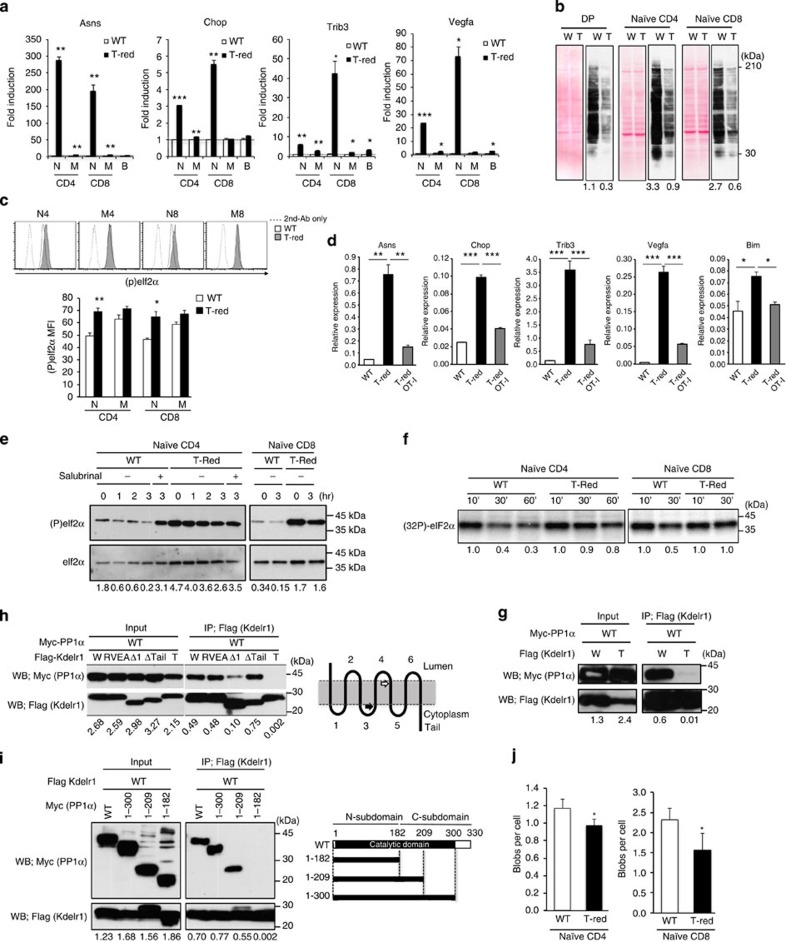
ISR are enhanced in T-Red naïve T cells due to a functional defect of PP1. (**a**) ISR target genes in T-cell subsets and B cells were examined by qPCR. N, M and B indicate naïve, memory and B cells, respectively. Expression levels of WT populations were normalized as 1. Data represent the mean+s.d. (*n*=2). (**b**) Global translation in DP thymocytes (DP), naïve T cells from WT (W) or T-Red (T) mice was examined by *in vivo* puromycin labelling (right). Ponceau-S staining is shown on the left. Numbers below the blots represent the intensity ratio of puromycin/Ponceau-S. (**c**) Phosphorylated-eIF2α in T-cell subsets were examined by flow cytometry. Representative FACS histograms from three independent experiments (top) and mean fluorescence intensity (MFI; bottom) are shown. Data represent the mean+s.e.m. (*n*=3). (**d**) ISR target genes in naïve CD8 T cells from WT, T-Red or OT-I/T-Red mice were examined. Relative expressions to *Hprt* levels are shown. Data represent the mean+s.d. (*n*=2). One-way ANOVA with *post hoc* Dunnett's test was used. (**e**) Naïve T cells were cultured and examined for phosphorylated-eIF2α levels. In some cultures, PP1/GADD34 inhibitor salubrinal was added. (**f**) Radiolabelled, phosphorylated-eIF2α was incubated for the indicated times with cell lysates from WT or T-Red naïve T cells, followed by SDS–PAGE and phosphor imaging. The intensity at 10 min in each group was normalized as 1.0. (**g**) Flag-KDELR1 WT or T-Red mutants and Myc-WT PP1α were overexpressed in HEK293T cells and immunoprecipitated with anti-Flag beads, followed by blotting with anti-Flag or anti-Myc. (**h**) Association of KDELR1 mutants with WT PP1α. A schema of KDELR1 is shown to the right. Closed and open arrows indicate the location of RVEA and T-Red mutations, respectively. (**i**) Association of PP1α mutants with WT KDELR1. A schema of PP1α is shown to the right. Numbers below the blots in **g**–**i** represent the ratio of PP1α/KDELR1. (**j**) Association of KDELR1 and PP1α in naïve T cells examined by proximity ligation assay (PLA). Numbers of association signals per cell are shown. Data represent the mean+s.d. Representative images from more than three independent experiments are shown in **b**,**d**,**f**–**h** and **i**. Mice between 7 and 13 weeks old were used. *P<0.05; **P<0.01; ***P<0.001.

We next attempted to identify how the apoptotic pathway is activated in T-Red naïve T cells. We performed DNA microarray analysis using freshly isolated naïve CD4+ and CD8+ T cells from WT and T-Red mice. Because Bim is known to be a target of ISR^11^, we investigated this pathway. Several genes known to be involved in ISR were also upregulated in T-Red naïve T cells. Quantitative PCR analysis confirmed that ISR-related genes, including *Asns*, *Chop*, *Trib3* and *Vegfα*, were significantly upregulated (Fig. 6a). Activated/memory T cells and B cells from T-Red mice also showed the upregulation of some stress genes, but the induction levels were much lower than in naïve T cells (Fig. 6a and Supplementary Fig. 5). In addition, activation of ISR is predicted to globally reduce translation in T-Red cells. Indeed, we found such a reduction in DP thymocytes and naïve T cells in T-Red mice (Fig. 6b).

A key pathway of ISR is mediated by eIF2α phosphorylation, which induces cell death when prolonged^12^,^13^. Phosphorylation of eIF2α was monitored by fluorescence-activated cell sorting (FACS) and western blotting and was found to be enhanced in T-Red naïve T cells (Fig. 6c and Supplementary Fig. 6a). Importantly, T-Red mice with TCR transgenic backgrounds, which have almost normal numbers of naïve T cells (Fig. 4a–c,f), significantly decreased the upregulation of ISR-related genes (Fig. 6d). On the other hand, mitochondrial stress, which potentially increases intracellular Bim expression and shortens cell survival time, was not induced in naïve T cells or thymocytes in T-Red mice, since neither mitochondrial membrane depolarization nor the expression of *Clpp* and *mtHSP60*, two target genes of mitochondrial stress^33^,^34^, were significantly induced (Supplementary Fig. 6b,c). Thus, these results suggest that ISR, but not mitochondrial stress, increased in T-Red naïve T cells.

### Dephosphorylation of eIF2α by PP1 is impaired

Since phosphorylation of eIF2α increased in T-Red naïve T cells, we focused on the regulation of eIF2α phosphorylation to understand how the ISR pathway is activated by KDELR1 dysfunction in T-Red naïve T cells. It is known that four kinases, PKR, PERK, GCN2 and haem-regulated eIF2α kinase, phosphorylate eIF2α and that PP1/GADD34 complexes dephosphorylate it^8^,^9^,^10^,^14^. Upstream events of the kinase activations, such as cellular ATP (indicative of glucose availability), reactive oxygen species (ROS) and iron levels^35^,^36^,^37^,^38^,^39^, were not specifically enhanced in T cells isolated from T-Red mice (Supplementary Fig. 7), suggesting the dephosphorylation of eIF2α by PP1 may be different. Naïve T cells showed a certain level of eIF2α phosphorylation even in WT mice (Fig. 6e). The phosphorylation of eIF2α was reduced after *in vitro* culture and reversed by the addition of a PP1/GADD34 specific inhibitor, salubrinal (Fig. 6e)^40^, suggesting that the decline of eIF2α phosphorylation levels was due to PP1 phosphatase activity. In T-Red naïve T cells, however, phosphorylation of eIF2α was prolonged, and almost no effect of salubrinal was evident, suggesting a functional defect of PP1/GADD34 phosphatase (Fig. 6e). Importantly, *in vitro* dephosphorylation assays showed that the phosphatase activity of phospho-eIF2α was impaired in T-Red naïve CD4+ and CD8+ T cells (Fig. 6f). In addition, only a negligible difference in PP1 defects, which were monitored by the dephosphorylation rates of eIF2α, was detected between WT CD4+ and WT CD8+ T cells (Fig. 6f). These results suggest that prolonged activation of eIF2α induced by dysfunctional PP1 phosphatase caused ISR in T-Red naïve T cells.

### Changes in KDELR1 association with PP1

PP1 is known to form complexes with various molecules to determine the PP1 enzyme activity, substrate specificity and subcellular localization^41^. We hypothesized that KDELR1 could be one such partner molecule and therefore investigated the association between KDELR1 and PP1 molecules. As shown in Fig. 6g, PP1α was co-immunoprecipiated with WT KDELR1. Importantly, the association of PP1α with the T-Red KDELR1 was substantially weaker than that with WT KDELR1 (Fig. 6g). KDELR1 contains a specific amino-acid sequence, RVEF, which matches a typical PP1-binding motif, RVXF^42^. However, we found that a loss-of-function mutation in the motif^42^ did not affect KDELR1-PP1α binding (Fig. 6h). Instead, cytoplasmic loop 1 was involved in the association, but the tail domain, which is known to contribute to the binding of ARF, GAP and Src family kinases^6^,^7^,^43^, was not (Fig. 6h). We also determined the region of PP1α responsible for the association. The C-subdomain of PP1α, which contains the binding sites for PP1 partners, including Inhibitor-1 and DARPP-32 (ref. 44), was not required for association with KDELR1 (Fig. 6i). Further truncation of PP1α abolished the association, suggesting that the amino-acid region 182–209 in the C-subdomain is responsible (Fig. 6i). These results support the idea that KDELR1 regulates PP1 activity via direct association.

Moreover, we found that KDELR and PP1 are associated in naïve T cells and that the degree of association was lower in T-Red naïve CD4+ and naïve CD8+ T cells compared with WT naïve T cells (Fig. 6j). Together with the reduction of T-Red naïve CD4+ and naïve CD8+ T cells, these results suggest that KDELR–PP1 association regulates naïve T-cell survival. Overall, our findings suggest that KDELR–PP1-mediated ISR regulation is involved in peripheral T-cell homeostasis.

## Discussion

It has been long thought that the primary function of KDELR is the retrograde transport of ER chaperones from the Golgi complex to the ER^1^,^2^. More recently, it has been found that KDELR also functions by activating Src family kinases on the Golgi complex^6^,^7^. Here we identify a new role for KDELR that affects naïve T-cell homeostasis *in vivo*—decreasing ISR-mediated cell death via the direct control of PP1 activity.

We established an ENU-induced mutant mouse strain that has a low number of naïve T cells (T-Red mice), finding this phenotype resulted from a point mutation in the *Kdelr1* gene. This point mutation caused an amino-acid substitution of KDELR1 at Ser123 to proline (Fig. 2b,c). A previous biochemical study revealed that a mutation at KDELR1 Ser123 abrogates ligand binding, possibly due to conformational alterations^45^. In addition, breeding analysis indicated that the T-Red phenotype is a recessive trait and that mutant mice with *Kdelr1* deficiency have a phenotype comparable to T-Red mice (Fig. 2e and Supplementary Fig. 2). Thus, we concluded the S123P point mutation causes dysfunctional KDELR1 in the T cells of T-Red mice and that the T-Red phenotype corresponds to the physiological function of KDELR1 molecules, at least in T cells.

Mechanistic analysis demonstrated that KDELR1 directly regulates PP1 activity, which is critical for preventing ISR in naïve T cells. T-Red KDELR1 association with PP1 was negligible, and its dephosphorylation activity against eIF2α was diminished in T-Red naïve T cells. In other words, dysfunctional KDELR1 may be unable to regulate eIF2α activation after the induction of various stresses related to ISR in T cells. It was reported that prolonged activation of eIF2α leads to apoptosis in cell lines *in vitro*^12^ and in neurons *in vivo*^13^. Consistent with these observations, T-Red naïve T cells showed an upregulation of genes encoding *Bim*, *Chop* and *Trib3*, all target genes and sometimes effector genes of the ISR pathway. Taken together, dysregulation of PP1 by the KDELR1 T-Red mutation induces ISR-mediated upregulation of proapoptotic factors including Bim, which may cause apoptosis in naïve T cells.

What is the relationship between the TCR-mediated signal and ISR? TCR transgenic data suggest that these transgenic TCR-mediated signals might mainly transduce a positive survival signal in naïve T cells in a manner dependent on TCR affinity for endogenous peptides (on MHC molecules) at steady state. Therefore, it is possible that a strong TCR signal rescues T-Red naïve T cells from ISR-mediated apoptosis by enhancing the activation of PP1 followed by the dephosphorylation of eIF2α. On the other hand, we also suggest that the reduction of naïve T cells is induced by their longer lifetime in the periphery most likely due to an accumulation of T-cell social stress.

Because we found that the numbers of DP and SP cells in T-Red OT-I mice are not reduced (Fig. 4d,e), we also hypothesize that TCR transgenic thymocytes transduced a relatively strong TCR signal from endogenous antigens compared with non-transgenic thymocytes just like naïve T cells are rescued from an excess ISR. Therefore, the transgenic thymocytes were negligibly affected even by the enhanced ISR in T-Red mice due to the excessive survival signal from the transgenic TCR. On the other hand, non-transgenic thymocytes that transduced a low TCR signal might be sensitive to the enhanced ISR in T-Red background, while those cells that have a high TCR signal were not. We, however, believe that the signalling events during the thymic selections are more complex compared with those in peripheral T cells. Therefore, further investigation is needed to answer this hypothesis particularly by using other high and low affinity TCR transgenic mice.

It remains unclear why proximal TCRα rearrangements were favoured in T-Red cells. One possibility is that proximal TCRα rearrangements make the time length of the DP stage in T-Red mice short to prevent ISR-mediated apoptosis induction, as it was reported that a longer presence of DP thymocytes corresponds with more use of distal TCRα rearrangements^46^. In other words, DP thymocytes, which rapidly differentiate to the SP stage, elude apoptosis, which is enhanced in T-Red thymocytes. We hypothesize that the longer DP stage could be more stressful to induce ISR, just like in peripheral naïve T cells. Consistent with this notion, the number of DP thymocytes that have TCR-rearrangements was reduced in T-Red mice compared with the number in WT mice (Fig. 1d). Moreover, an inducer of double strand breaks, etoposide, caused more death of T-Red DP thymocytes than that of WT thymocytes (Supplementary Fig. 8). Thus, it is possible that the shorter length of the DP thymocyte stage in T-Red mice may explain why proximal TCRα rearrangements are favoured in T-Red cells.

How the KDELR1-PP1 association regulates PP1 activity is a matter of future study. PP1 is known to have many regulatory proteins that determine phosphatase activity, substrate specificity and subcellular localization^41^, including the molecular chaperone Bip^47^,^48^, which has the KDEL motif and binds to KDELR1 (ref. 49). It was also reported that PP1/GADD34 complexes are localized to the ER^50^, where KDELR1 transports chaperones from Golgi compartments. We suggest then that KDELR1 may supply certain molecules, such as KDELR-binding chaperones that associate with PP1 and change the PP1 structure to dephosphorylate eIF2α efficiently. Moreover, it is also possible that a strong TCR signalling might directly increase KDELR1 activity or reduce ISR response.

In summary, we generated an ENU mutant strain with a reduced number of naive T cells, which we named T-Red. Positional cloning and subsequent experiments identified the gene responsible for this effect as KDELR1. Mechanistic analysis suggested that KDELR1 regulates ISR in naïve T cells. Phosphorylation of eIF2α, a central checkpoint of ISR, is enhanced because activity of a key phosphatase of this pathway, PP1, is reduced in T-Red naïve T cells. T-Red KDELR1 did not associate with PP1, a property that correlated with reduced phosphatase activity. Indeed, naïve T cells in T-Red mice increased the expression of several target genes in the eIF2α pathway, including *Bim*, *Chop* and *Trib3*, and caused apoptosis. We also hypothesize that physiological high-affinity TCR signal regulates T-Red KDELR1-mediated excess ISR in naïve T cells. We suggest that KDELR1 regulates ISR by controlling PP1 activity to maintain naïve T-cell homeostasis *in vivo*.

## Methods

### Mouse strains

C57BL/6 mice and C3H/He mice were purchased from Charles River Japan. All mice were maintained under specific pathogen-free conditions according to the protocols of Osaka University Medical School and RIKEN Research Center for Allergy and Immunology (RCAI). Mice of both sexes were used. The age of mice is indicated in figure legends. There are no sample exclusion criteria. No randomization or blinding was used. Sample size of more than three mice was chosen to ensure power for Student's *t*-test unless the availability of mice was limited. All animal experiments were performed following the guidelines of the Institutional Animal Care and Use Committees of the Graduate School of Frontier Bioscience and Graduate School of Medicine, Osaka University; the Institute for Genetic Medicine and Graduate School of Medicine, Hokkaido University; and RIKEN RCAI.

### Antibodies and reagents

The following antibodies were used for FACS staining at 200-fold dilution except for anti-CD90 antibodies, which were diluted to 2,000-fold: eFlour450-conjugated anti-CD4 (RM4-5), anti-CD8 (53-6.7) and anti-B220 (RA3-6B2) (eBioscience, San Diego, California); BV421-conjugated anti-CD19 (6D5) (BioLegend, San Diego); FITC-conjugated anti-CD44 (IM7) (eBioscience) and anti-IgD (11-26c.2a) (BD Biosciences, San Jose, California); PE-conjugated anti-CD25 (PC61), anti-CD45.2 (104), anti-CD62L (MEL14), anti-IL-17A (eBio17B7) (eBioscience), anti-IgM (R6-60.2) and anti-IgG1 (A85-1) (BD Biosciences); PE-Cy7-conjugated anti-CD3 (145-2C11) (BioLegend), anti-CD8 and anti-CD44 (eBioscience); APC-conjugated anti-CD4, anti-CD45.1 (A20), anti-CD90.1 (HIS51), anti-CD90.2 (53-2.1), anti-interferon (IFN)-γ (XMG1.2) (eBioscience), and anti-CD19 (BioLegend); biotin-conjugated CD273 (TY25) (BioLegend); anti-Bim (Cell Signaling, Tokyo, Japan); and Alexa488-conjugated anti-rabbit IgG (Invitrogen, Tokyo). Antibodies for western blotting were as follows: anti-Bim and anti-phospho S51 eIF2α (Cell Signaling); anti-FLAG M2 affinity gel and 3xFLAG peptide (Sigma, Tokyo); and anti-eIF2α, anti-Actin, anti-PP1 and HRP-conjugated anti-cMyc (Santa Cruz, Dallas, Texas). They were used at 100-fold dilution.

### Flow cytometry and cell sorting

For cell surface labelling, ∼10^6^ cells were incubated with fluorescence-conjugated antibodies for 30 min on ice in the presence of non-labelled anti-CD16/32 (2.4G2) antibody for blocking. Intracellular staining was performed using the Cytofix/Cytoperm kit (BD Biosciences), or the Foxp3 Fixation/Permeabilization kit (eBioscience) for phosphorylated eIF2α staining. Cells were then analysed with the CyAn flow cytometer (Beckman Coulter, Tokyo). Collected data were analysed using Flowjo software (Tree Star, Ashland, Oregon). To purify naïve and memory T cells, splenocytes and lymph node cells were sorted based on their CD44 expression levels using the Moflo cell sorter (Beckman Coulter). CD4^+^CD8^+^CD25^−^ thymocytes were sorted as DP thymocytes. IgM^+^CD273^−^ B cells were sorted as naïve IgM^+^ B cells^51^,^52^. Cell purity was routinely >98%. Antibody dilutions were 100-fold and 200-fold for intracellular staining and cell sorting, respectively.

### Establishment of ENU mutant mice and SNP analysis

ENU (Sigma) was injected intraperitoneally (i.p.) into B6 male mice twice at 1-week interval^53^. After a sterile period (10 to 11 weeks), these G0 male mice were mated with female B6 mice to generate G1 populations. G2 populations were made by artificial insemination using G1 sperm and normal B6 eggs. G3 offspring were generated by G2 intercrosses. About 30 G3 mice per G1 pedigree were checked for T-cell populations in peripheral blood. A mutant mouse was crossed with WT B6 to establish a mutant mouse line. For linkage analysis, T-Red mice (B6 background) were crossed with C3H/HeJ mice, and F1 mice were intercrossed. F2 populations were checked for CD44 levels on CD8 T cells in peripheral blood. The region responsible for the T-Red phenotype was determined by SNP analysis using tail DNA from F2 mice with high CD44 levels in T cells.

### Retroviral transduction of *Kdelr1*

B6 or T-Red mice were injected i.p. with 150 mg kg^−1^ of 5-fluorouracil (Sigma, Tokyo) 3 to 5 days before harvesting bone marrow cells. Bone marrow cells were cultured for 2 days in the presence of 100 ng ml^−1^ IL-6, 100 ng ml^−1^ stem cell factor and 1% IL-3-conditioned medium. Phoenix cells were transfected with pMSCV-IRES-GFP KDELR1 or mock vectors, and virus-containing medium was collected after 2 days. Spin infection was performed twice in the presence of 4 mg ml^−1^ polybrene (Sigma). The resulting transduced bone marrow cells were transplanted into 9.5 Gy-irradiated B6 or T-Red mice. Recipient mice were analysed 8–12 weeks later.

### Establishment of *Kdelr1*-deficient mice

KDELR1 conditional knockout mice were generated by a conventional homologous recombination technique in ES cells. The targeting vector was constructed in a pEZ-FRT-Lox-DT vector so that the second and third exons of the *Kdelr1* gene were flanked with loxp sites (Supplementary Fig. 9). The FRT-site flanked neomycin-resistant gene was removed by crossing with flippase transgenic (Tg) mice. *Kdelr1*^flox/flox^ mice were then crossed with CAG-cre Tg or CD4-cre Tg mice to generate conditional *Kdelr1* knockout mice.

### *In vivo* experiments

Collagen-induced arthritis was induced using a *Mycobacterium bovis* Bacillus Calmette-Guérin cell wall skeleton (BCG-CWS) emulsified in CFA^54^. Chicken type II collagen (Sigma Aldrich) was injected with CFA/BCG-CWS at 200 μg per mouse at tail base on days 0 and 21. Serum IL-17 was measured using an IL-17 ELISA (enzyme-linked immunosorbent assay) kit (eBioscience). The anti-OVA response was elicited by i.p. injection of alum-precipitated OVA on days 0 and 5. Serum anti-OVA IgG1 and IgM levels were measured by ELISA in which an OVA-coated microtiter plate and alkaline phosphatase-conjugated anti-mouse IgG1 or IgM antibodies (Jackson Immunoresearch, Philadelphia, Pennsylvania) were used. T-cell responses to male-specific antigens were induced by injection of live splenocytes from male B6 mice (2 × 10^7^ cells) into congenic female B6 mice (CD45.1^+^)^55^. The donor CD45.2^+^ population in peripheral blood was examined over time. The detection limit was determined using anti-CD45.2 staining of blood leukocytes from B6.SJL mice. Bacterial infection using *Listeria monocytogenes* expressing OVA (LM-OVA) was performed as described^56^. In brief, mice were infected intravenously with 3,000 colony-forming units LM-OVA on day 0. In some experiments, congenically marked recipients received 10,000 to 20,000 WT or T-Red OT-I cells 1 day before infection. On day 7, the OT-I population was checked by flow cytometry. The antigen-specific IFN-γ-secreting CD8+ T-cell population was determined by *in vitro* stimulation of splenocytes with OVA peptide (N4, SIINFEKL) for 4 h followed by intracellular IFN-γ staining. Mixed bone marrow chimera mice were prepared by transplanting a mixture of T-cell-depleted bone marrow cells from WT (CD45.2/CD90.1), T-Red (CD45.1/CD45.2/CD90.2), OT-I (CD45.2/CD90.1/CD90.2) and T-Red/OT-I (CD45.2/CD90.2) mice into lethally irradiated (10 Gy) WT congenic mice (CD45.1/CD90.2). The ratio of the mixture was 4:4:1:1 to conform with a polyclonal situation. For *in vivo* survival assay of naïve T cells, equal numbers of sorted naïve CD4 or naïve CD8 T cells from WT and *Kdelr1* flox;ERT2-cre mice were mixed and transferred into congenic mice on day 0. Tamoxyfen (1.5 mg per mouse, p.o.) was administered on days 1, 3 and 8. The frequency of donor cells in blood was examined on days 1, 14 and 21 after donor cell transfer, and the absolute numbers of donor cells in the spleen were examined on day 21.

### Metabolic labelling using puromycin

A non-radioactive method was employed to monitor protein synthesis^57^,^58^. In brief, mice were injected i.p. with puromycin (Sigma) at 20 mg kg^−1^. After 1 h, splenocytes and thymocytes were harvested and sorted for naïve T cells and DP thymocytes. Total cell lysate was subjected to western blotting using anti-puromycin antibody at 8,000-fold dilution (clone 4G11, Millipore, Billerica, MA), followed by Ponceau-S staining of the membrane. Densitometry analysis was performed using ImageJ software (National Institutes of Health, Bethesda, Maryland).

### *In vitro* T-cell culture

Sorted naïve or memory T cells (1 × 10^5^ cells) were cultured in a 96-well plate in the presence or absence of 2.5 ng ml^−1^ recombinant mouse IL-7 (Peprotech, Rocky Hill, New Jersey). Cells were stained with 7-AAD (eBioscience) and analysed by a CyAn flow cytometer (Beckman Coulter). The living cell population was determined as 7-AAD negative. In some experiments, naïve T cells or DP thymocytes were irradiated at 0.5 Gy or cultured with etoposide (Sigma) at 1 μg ml^−1^. For detection of eIF2α, sorted T cells that were freshly isolated or preincubated at 37 °C for 1–2 h to reduce the endogenous level of eIF2α phosphorylation were lysed in RIPA buffer, and total cell lysate was subjected to Western blotting. In some experiments, salubrinal (Santa Cruz), a PP1/GADD34 specific inhibitor, was added at 10 μM after the preincubation. For Th1 or Th17 cell differentiation, sorted naïve CD4 T cells were cultured for 4-5 days with bone marrow-derived dendritic cells and soluble anti-CD3ɛ mAb (145-2C11) in the presence of IL-12 (for Th1) or IL-6 and TGF-β (for Th17). Intracellular cytokine staining was performed using fluorescence-labelled anti-IFN-γ and anti-IL-17A at 200-fold dilutions (eBioscience).

### Real-time PCRs

The 7,300 real-time PCR system (ABI, Tokyo) and SYBR FAST PCR Mix or PROBE FAST PCR Mix (KAPA Biosystems, Woburn) were used to quantify the expression levels of each gene and HPRT mRNA. Total RNA was prepared from cell-sorted, purified T cells using the GenElute Mammalian total RNA kit (Sigma). The conditions for real-time PCR were 40 cycles at 94 °C for 15 s followed by 60 °C for 60 s (SYBR green), or 40 cycles at 94 °C for 3 s, followed by 60 °C for 30 s (FAM and TAMRA dual-labelled probes (Sigma)). Relative mRNA expression levels were normalized to the levels of *Hprt* mRNA. qPCR primer sequences are: *Bim*, 5′FAM-TGAACTCGTCTCCGATCCGCCGCA-TAMRA3′, 5′-ACGACAGTCTCAGGAGGAACC-3′ and 5′-CGGTAATCATTTGCAAACACCCTC-3′; *Chop*, 5′FAM-TCTTGACCCTGCGTCCCTAGCTTGGC-TAMRA3′, 5′-CCCAGGAAACGAAGAGGAAGAA-3′ and 5′-GGGATGTGCGTGTGACCTC-3′; *Hprt*, 5′FAM-ATCCAACAAAGTCTGGCCTGTATCCAACAC-TAMRA3′, 5′-AGCCCCAAAATGGTTAAGGTTG-3′ and 5′-CAAGGGCATATCCAACAACAAAC-3′; *Asns*, 5′-GGCCCTGGATGAAGTCATATT-3′ and 5′-CACCACGCTGTCTGTGTTCT-3′; *Vegfa*, 5′-TCACCAAAGCCAGCACATAG-3′ and 5′-AATGCTTTCTCCGCTCTGAA-3′; and *Trib3*, 5′-GCCTTATATCCTTTTGGAACGA-3′ and 5′-AGATGTAAAGGAGCCGAGAGC-3′.

### Immunoprecipitation and western blotting

HEK293T cells were transfected with a calcium phosphate transfection method. The pBOS-EF expression vector with Myc or FLAG tag was used for forced expression. Total cell lysate was immunoprecipitated with anti-FLAG M2 affinity gel (Sigma, Tokyo). The 3 × FLAG peptide (Sigma) was used to elute the immunoprecipitated fraction. SDS–polyacrylamide gel electrophoresis (SDS–PAGE) and western blotting was performed by standard methods^27^. Densitometry analysis was performed using ImageJ software. The original gel images are shown in Supplementary Fig. 10.

### TCR Jα and TCRβ usage/recombination

Total RNA was extracted from DP thymocytes, and cDNA was synthesized using oligo dT. TCR Vα8-Cα fragments were amplified by PCR and blotted with ^32^P-labelled Jα specific oligonucleotide probes^59^. Hybridization was performed overnight at 50 °C, and washing was carried out for 20 min at 60 °C twice in 6 × SSC/0.1% SDS. Primer and probe sequences are shown in Supplementary Table 1. TCR Vβ usage was examined by flow cytometry using the Vβ TCR screening panel (BD Biosciences). *Cβ1* mRNA levels were measured by qPCR using SYBR green. TCR Dβ2-Jβ2 recombination was examined by genomic DNA PCR using primers listed in Supplementary Table 1. The insulin gene was used as a positive control for genomic DNA amplification^60^.

### Dephosphorylation assay of eIF2α

A dephosphorylation assay for eIF2α^61^,^62^ was performed using naïve T-cell lysates. Sorted naïve T cells were lysed in a cell lysis buffer containing 20 mM Tris-HCl, pH 7.4, 0.5% Triton-X-100, 50 mM NaCl, 10% glycerol and 0.1 mM EDTA. Recombinant GST-PERK and His-eIF2α (Sigma) were incubated for 30 min at 30 °C in the presence of 5 μCi [γ-^32^P]ATP. After removal of unincorporated isotopes, a portion of the radiolabeled proteins was incubated at 30 °C with 5 ml of total T-cell lysate in a 10-μl reaction (dephosphorylation buffer; 20 mM Tris-HCl, pH 7.4, 50 mM KCl, 2 mM MgCl_2_, 0.1 mM EDTA, 0.8 mM ATP). The reaction was stopped by adding 5 × SDS–PAGE sample buffer followed by boiling and SDS–PAGE. Images were obtained using Typhoon Phosphorimager (GE Healthcare, Tokyo). Densitometry analysis was performed using ImageJ software.

### Measurement of mitochondria stress responses

Mitochondrial membrane depolarization was examined using the MitoPT JC-1 kit (ImmunoChemistry Technologies, Bloomington, Minnesota). Total thymocytes were stained with JC-1 dye followed by cell surface staining. A shift of fluorescence was detected by flow cytometry. As a positive control of the JC-1 staining, thymocytes were incubated with carbonylcyanide *m*-chlorophenylhydrazone at 50 μM for 30 min. The expressions of *Clpp* and *mtHSP60*, two target genes for mitochondrial stress^33^,^34^, were examined by real-time PCR.

### Measurement of ATP/ROS/iron levels

Intracellular ATP levels were measured using a commercially available luciferase-based kit (Toyo B-Net, Tokyo). For ROS detection, splenocytes were stained with 2 μM H_2_D-CFDA for 15 min at 37 °C followed by cell surface staining for flow cytometry. Iron levels were examined using calcein-AM (Sigma)^63^. Splenocytes were stained with 0.025 μM calcein-AM for 30 min at 37 °C followed by cell surface staining for flow cytometry. Binding of intracellular iron to calcein quenched the calcein fluorescence.

### Proximity ligation assay

PLA was performed to examine KDELR1 and PP1 association at endogenous expression levels. Sorted naïve T cells from WT and T-Red mice were seeded in a microscopy chamber (Ibidi), then fixed and permeabilized using the Cytofix/Cytoperm kit (BD Biosciences). After incubation with anti-KDELR1 and anti-PP1 (Santa Cruz) at 100-fold dilutions, a PLA reaction was performed using a commercially available kit (Duolink, Sigma). Blob-like fluorescent PLA signals were obtained by Z-stack imaging using confocal microscopy. The number of blobs was automatically counted in more than 200 cells by computer software.

### Statistical analysis

Student's *t*-test (two-tailed) was used for the statistical analysis of differences between two groups unless stated otherwise. For multiple comparisons, one-way ANOVA (analysis of variance) and *post hoc* Dunnett's test were used. A paired Student's *t*-test was used for some experiments. Wilcoxon test was used for the evaluation of arthritis clinical scores. *P* values <0.05 were considered statistically significant.

## Additional information

**How to cite this article:** Kamimura, D. *et al.* KDEL receptor 1 regulates T-cell homeostasis via PP1 that is a key phosphatase for ISR. *Nat. Commun.* 6:7474 doi: 10.1038/ncomms8474 (2014).

## Supplementary Material

Supplementary Information Supplementary Figures 1-10, Supplementary Table 1 and Supplementary References

## Figures and Tables

**Table 1:** T-Red phenotype is autosomal recessive.

**Parents**	**Generation**	***n***	**Memory phenotype**	**%**
G3.mutant × G3.mutant	G4	28	28	100
B6 × G3.mutant	F1	21	0	0
	F2	106	21	19.8

T-cell phenotypes in a mutant mouse strain were inherited as a simple autosomal recessive trait in the progeny. Peripheral blood of mice between 6 and 12 weeks old was examined for CD44 levels of CD8 T cells.
