# Supplementary material for: KDEL receptor 1 regulates T-cell homeostasis via PP1 that is a key phosphatase for ISR
Source: Nat Commun. 2015 Jun 17;6:7474. doi: 10.1038/ncomms8474 (PMC4557295; doi:10.1038/ncomms8474)
Supplement: Supplementary Information — Supplementary Figures 1-10, Supplementary Table 1 and Supplementary References [file ncomms8474-s1.pdf]

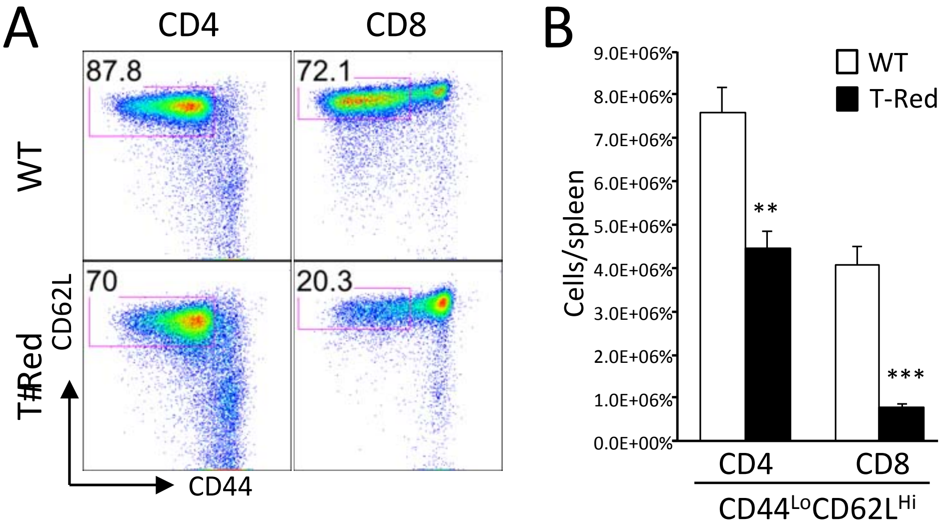

Supplementary Figure 1. Reduction of CD44lowCD62Lhigh naïve T cells in T-Red mice.

CD44 and CD62L plots of CD4 or CD8 T cells (A) and the absolute numbers in the spleen (B) are shown. CD4 or CD8 T cell population was gated on $CD19^{NEG}$MHC class $II^{NEG}CD3^{+}$. Percentages in CD44low CD62Lhigh naïve T cell population are shown in A. Mice between 7-9 weeks old were used. Data represent the mean ± SEM (n = 4). **, *P* < 0.01 and ***, *P* < 0.001.

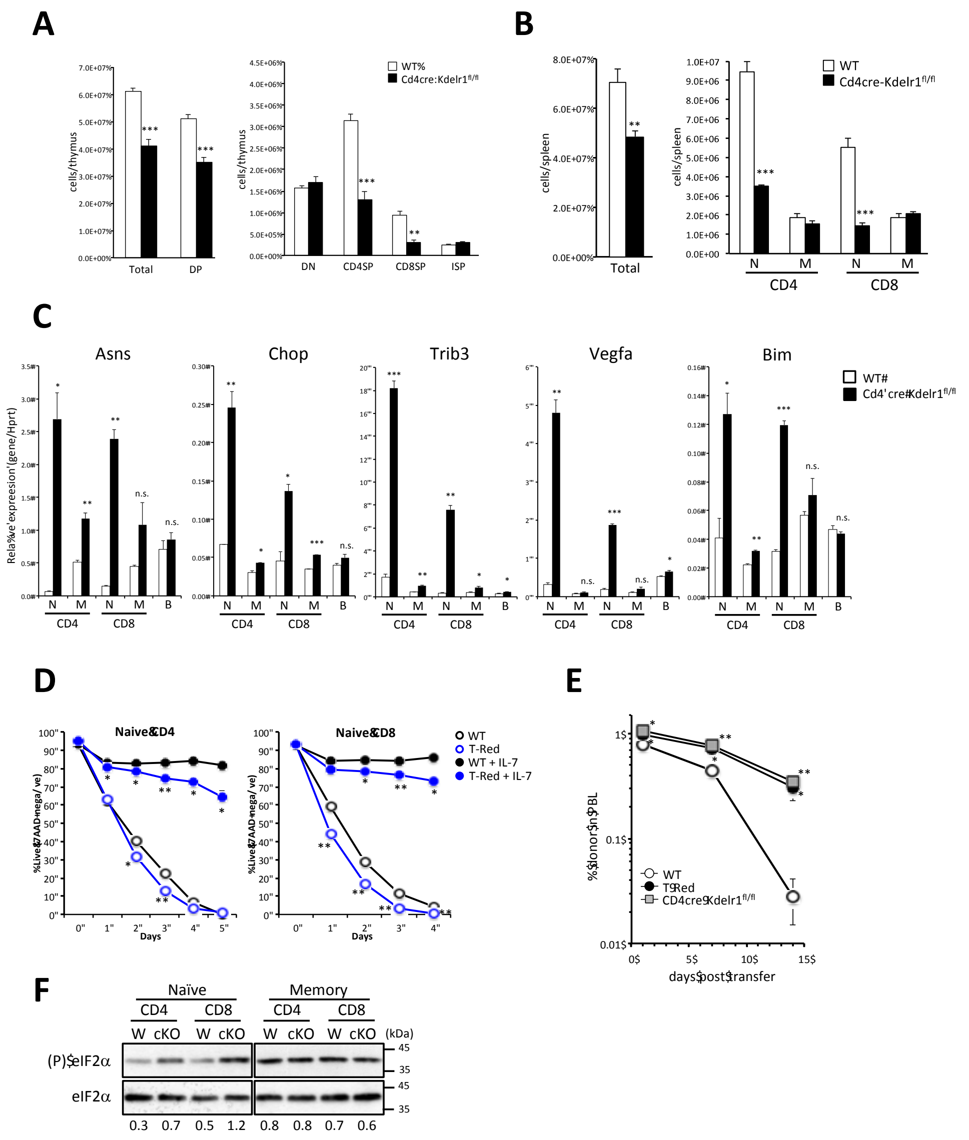

Supplementary Figure 2. T-cell specific *Kdelr1* deletion is equivalent to T-Red mice

(A, B) Thymic (A) and splenic (B) cell numbers in WT and CD4cre-Kdelr1$^{fl/fl}$ mice (6-11 weeks old) are shown. For thymic populations, DN, DP, CD4SP, CD8SP and ISP indicate CD4-CD8- double-negative, CD4+CD8+ double-positive, CD3highCD4+CD8- CD4 single-positive, CD3highCD4-CD8+ CD8 single-positive and CD3lowCD4-CD8+ immature single-positive populations, respectively. For splenic populations, N and M indicate naïve and memory T cells, respectively. Data represent the mean + SEM (n = 8-10). **, $P < 0.01$ and ***, $P < 0.001$. (C) mRNA levels for the ISR target genes and Bim in naïve CD4, memory CD4, naïve CD8 and memory CD8 T cells, and total B cells are shown. N, M and B indicate naïve, memory T cells, and B cells, respectively. Mice between 6-8 weeks old were used. Data represent the mean + SD (n = 2). *, $P < 0.05$; **, $P < 0.01$; and ***, $P < 0.001$. n.s., not significant. (D) The survival of naïve CD4 and CD8 T cells sorted from WT or T-Red mice (7-10 weeks old) was examined in vitro in the presence or absence of IL-7. Data represent the mean ± SD (n = 2). *, $P < 0.05$ and **, $P < 0.01$. (E) Male cell rejection in female mice. Congenic CD45.1+ male splenocytes were transferred into female WT, T-Red, or CD4cre-Kdelr1$^{fl/fl}$ mice (6-8 weeks old). Percentages of the transferred donor cells in peripheral blood leukocytes (PBL) are shown. Data represent the mean ± SEM (n = 3-4). *, $P < 0.05$ and **, $P < 0.01$. (F) Phosphorylated eIF2α levels were examined by immunoblotting naïve CD4, naïve CD8, memory CD4 and memory CD8 T cells sorted from WT (W) or CD4cre-Kdelr1$^{fl/fl}$ conditional KO (cKO) mice (6-8 weeks old). The intensity ratio of phosphorylated eIF2α□total eIF2α is shown below the blots. Representative images from 3 independent experiments are shown.

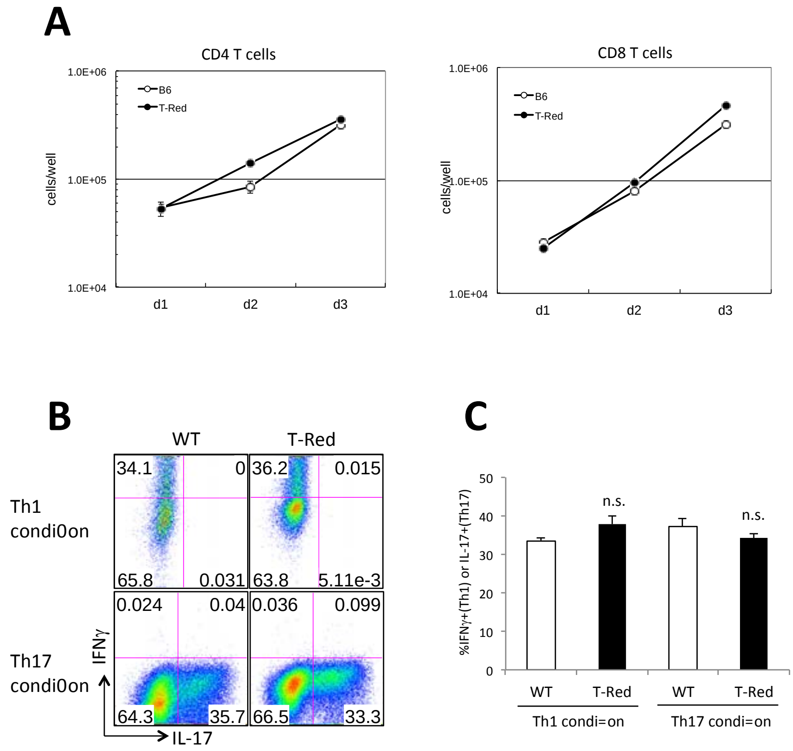

Supplementary Figure 3. Proliferation and Th1/Th17 differentiation are not impaired

(A) Naïve CD4 or CD8 T cells were sorted from WT or T-Red mice at 7 weeks old and cultured for the indicated days with anti-CD3 and anti-CD28 mAb stimulations. Cell numbers are shown. (B, C) Naïve CD4+ T cells were sorted from WT or T-Red mice at 6-8 weeks old and cultured in the Th1 or Th17 condition. Intracellular IL-17 and IFN-γ staining was performed on day 4. Representative FACS plots from 3 independent expriments (B) and the percentage of Th1 and Th17 differentiated populations (C) are shown. Data represent the mean + SD (n = 2). n.s., not significant.

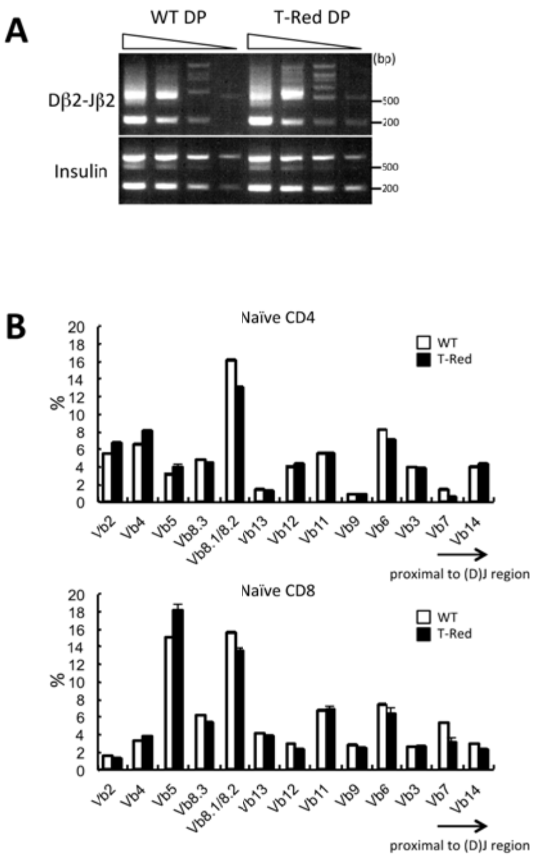

Supplementary Figure 4. TCR Dβ2-Jβ2 recombination and Vβ usage are not impaired

(A) The TCR Dβ2-Jβ2 region was amplified using serially diluted genomic DNA of double-positive (DP) thymocytes from WT or T-Red mice (8-12 weeks old). The insulin gene was used as a positive control for genomic DNA amplification. Multiple bands represent the Jβ2-1 to Jβ2-6 segments. Representative images from 2 independent experiments are shown. (B) TCR Vβ usage in splenic naïve CD4 or CD8 T cells of WT or T-Red mice (5-6 weeks old) was examined by flow cytometry. Percentages of each Vβ within naïve CD4 or CD8 T cell population are shown. Data represent the mean + SEM (n = 3-4). Unlike TCR Jα, no preferential usage of a Vβ chain proximal to the (D)J region, such as Vβ7 or Vβ14, was observed.

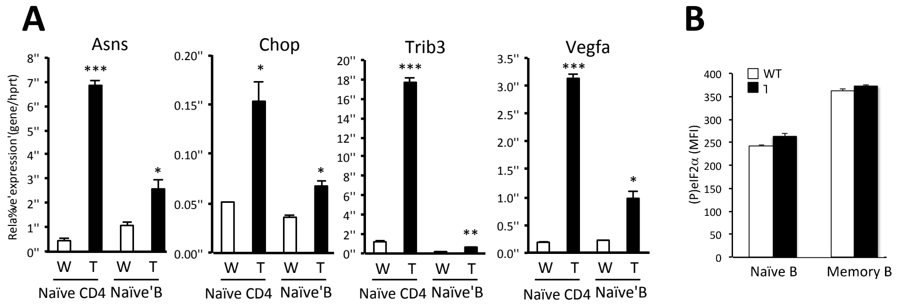

Supplementary Figure 5. T-Red naïve B cells show less ISR.

(A) mRNA levels of the ISR target genes in naïve CD4 T cells or IgM+CD273- naïve B cells (Naïve B) sorted from WT or T-Red mice (6-8 weeks old). Data represent the mean + SD (n =2). *, p < 0.05; **, p < 0.01; and ***, p < 0.001. (B) The mean fluorescence intensity (MFI) of phosphorylated eIF2α examined by flow cytometry is shown. Naïve B and memory B cells indicate IgM+CD273- and IgM+CD273+ B cells, respectively [5,6]. Mice between 9-11 weeks old were used. Data represent the mean + SEM (n = 3-4). *, *P* < 0.05.

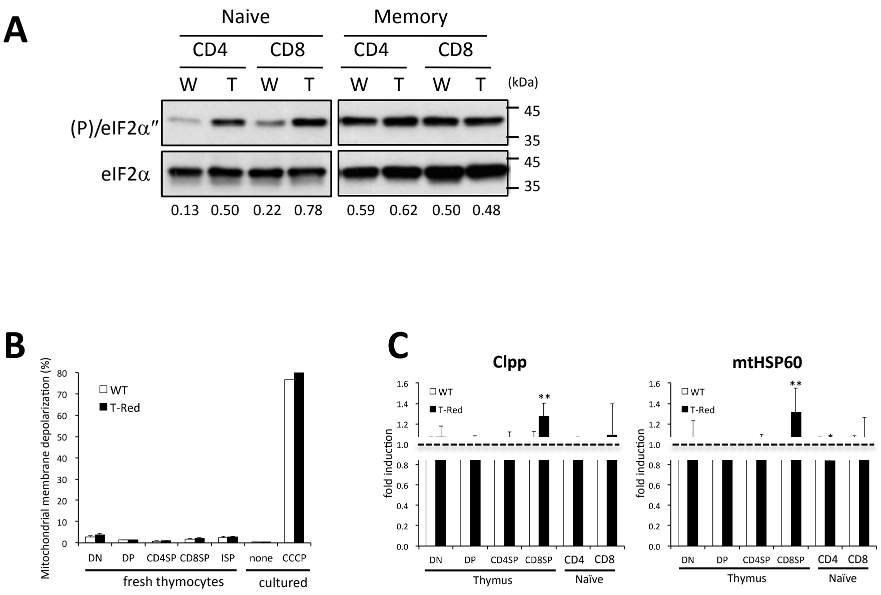

Supplementary Figure 6. WB of phosphorylated eIF2α and mitochondrial stress.

(A) Western blotting was performed for S51 phosphorylated and total eIF2α in T cell subsets sorted from WT (W) or T-Red (T) mice (6-9 weeks old). The intensity ratio of phosphorylated eIF2α/total eIF2α is shown below the blots. Representative images from 3 independent experiments are shown. (B, C) Mitochondrial stress responses. Mitochondrial membrane depolarization was examined by staining with JC-1 dye (B). Carbonylcyanide *m*-chlorophenylhydrazone (CCCP) was used as a positive control for the staining. mRNA levels of mitochondrial stress target genes were examined by real-time PCR (C). Expression levels of WT populations were normalized to 1. DN, DP, CD4SP, CD8SP and ISP indicate CD4-CD8- double-negative, CD4+CD8+ double-positive, CD3highCD4+CD8- CD4 single-positive, CD3highCD4-CD8+ CD8 single-positive and CD3lowCD4-CD8+ immature single-positive populations, respectively. Mice between 5-7 weeks old were used. Data represent the mean + SEM (B, n = 3) or SD (C, n = 2). *, $P < 0.05$ and **, $P < 0.01$.

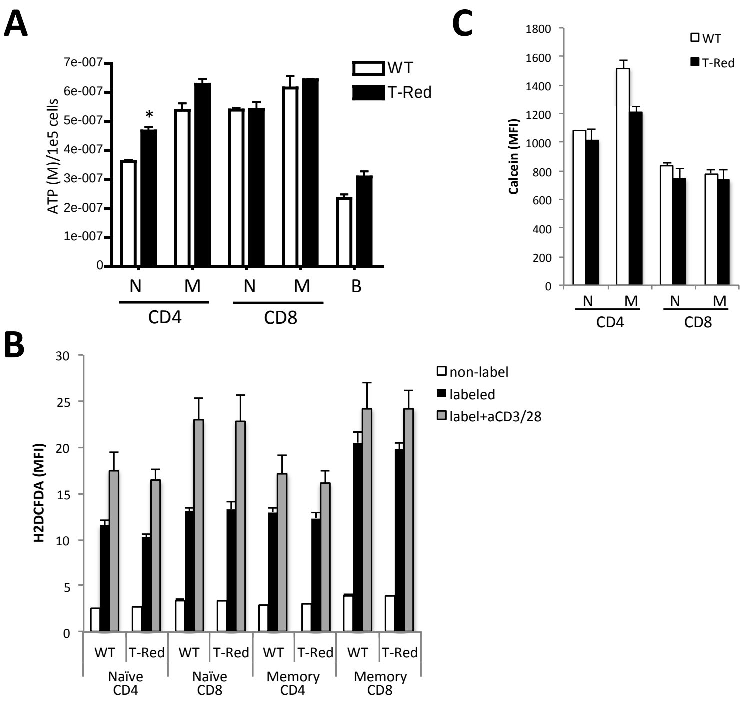

Supplementary Figure 7. Activation of ISR kinases is not implicated.

(A) Naïve CD4, naïve CD8, memory CD4 and memory CD8 T cells as well as B cells were sorted and had their ATP levels measured in cell lysates. N, M and B indicate naïve, memory T cells, and B cells, respectively. ATP was not reduced in T-Red T cells, suggesting these cells are not starved of energy resources like glucose, which would otherwise lead to the activation of GCN2, PKR and PERK kinases [1]. (B) Total splenocytes were stained with H_2D-CFDA and cell surface markers for T cells. ROS levels in each T cell population were determined by flow cytometry. The mean fluorescence intensities (MFI) of H_2D-CFDA non-labeled (open columns), H_2D-CFDA-labeled (closed columns), H_2D-CFDA-labeled and anti-CD3 and anti-CD28 mAb stimulated (gray columns) populations are shown. ROS levels, which potentially activate PERK and PKR kinases [2,3], were not increased in T-Red T cells. (C) Total splenocytes were stained with calcein AM and cell surface markers for T cells. Intracellular iron levels in each T cell population were determined by flow cytometry. The MFI of calcein is shown. Note that more iron results in less MFI because iron quenches calcein. Iron levels were not decreased in T-Red T cells. Iron deficiency causes HRI kinase activation followed by elF2α phosphorylation [4]. Mice between 6-11 weeks old were used for all experiments. Data represent the mean + SD (n = 2).

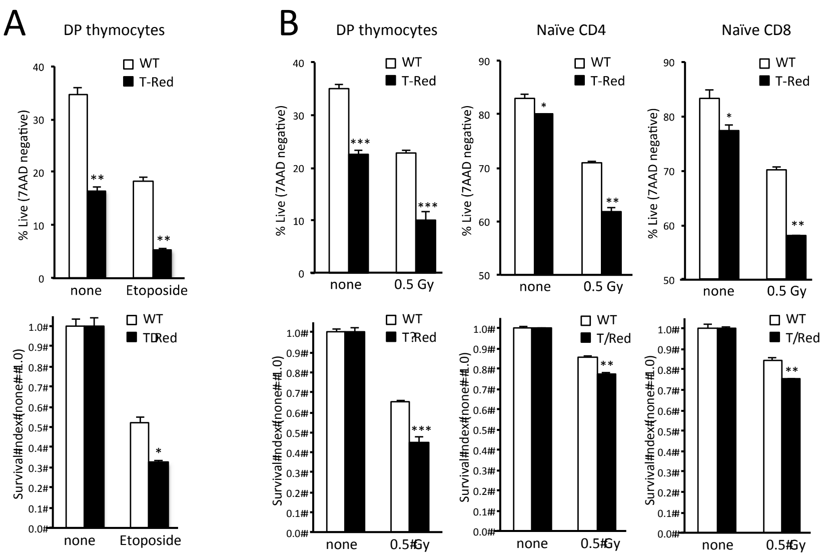

Supplementary Figure 8. T-Red naïve T cells are more susceptible to stressors.

(A) Double-positive (DP) thymocytes were sorted from WT or T-Red mice (8-9 weeks old) and cultured for 2 days in the presence of 1 μM etoposide. (B) Double-positive (DP) thymocytes, naïve CD4 and naïve CD8 T cells were sorted from WT or T-Red mice (8-10 weeks old) and irradiated at 0.5 Gy. These cells were cultured for 2 days. IL-7 was added to the naïve T cell culture. The percentage of the live cells is shown. Data represent the mean + SD (n = 2). *, $P < 0.05$; **, $P < 0.01$; and ***, $P < 0.001$.

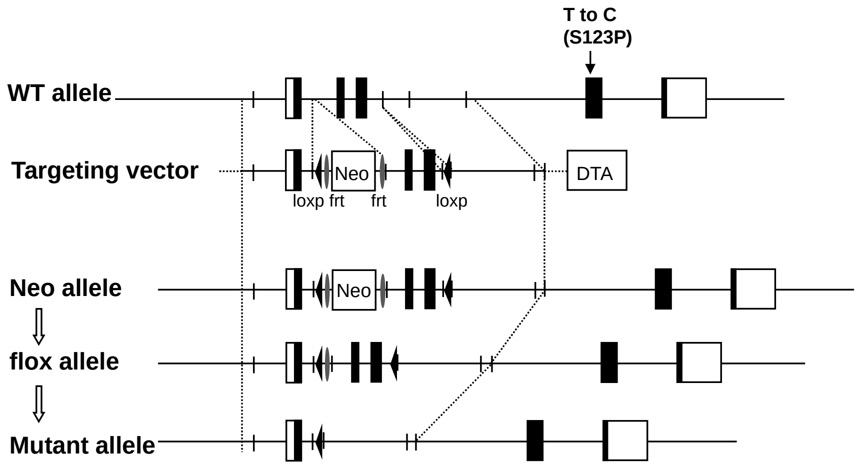

Supplementary Figure 9. Targeting construct of *Kdelr1* deficient mice.
The construct for *Kdelr1* deficient mice was designed to remove the second and third exons of the *Kdelr1* gene using the Cre-loxP system. The FRT-site-flanked neomycin-resistant gene was removed by crossing with flippase transgenic mice (see Neo allele to flox allele). Mice with the flox allele of *Kdelr1* were bred with CAG-Cre or CD4-Cre transgenic mice. For systemic deletion of the *Kdelr1* gene, mice homozygous for the mutant alleles without the CAG-Cre transgene were used.

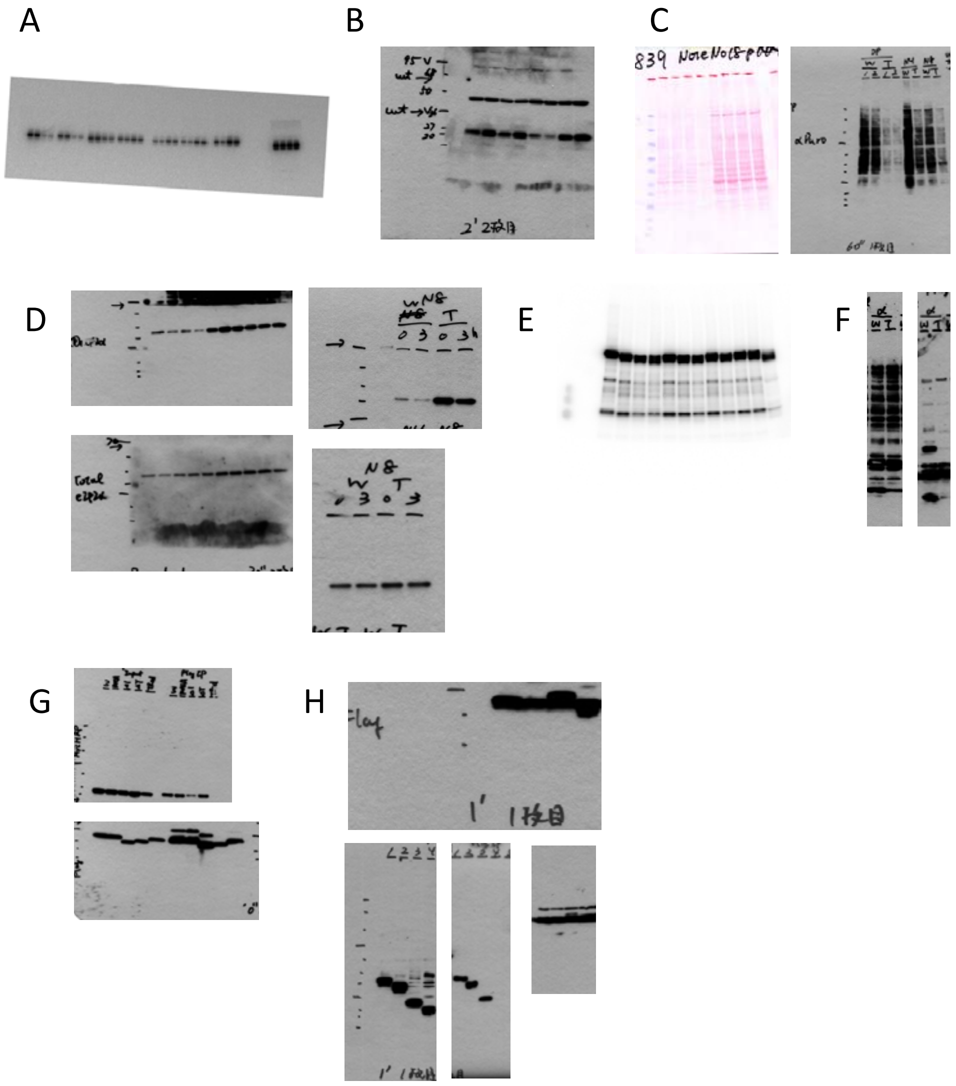

Supplementary Figure 10. The original images for the main figures.

(A) Fig. 4H, (B) Fig. 5B, (C) Fig. 6B, (D) Fig. 6E, (E) Fig. 6F, (F) Fig. 6G, (G) Fig. 6H, and (H) Fig. 6I.

Supplementary Table 1. Primers and probes used for TCR Jα and TCRβ usase

Name	Sequence
**Jα58**	ACTGGGTCTAAGCTGTCATTTGGG
**Jα53**	AGTGGAGGCAGCAATTACAAACTG
**Jα47**	CTTTGGCTTGGGAACCATTTTG
**Jα39**	AATGCAGGTGCCAAGCTCAC
**Jα27**	GACCGTGCTCACAGTGAAG
**Jα16**	TTAGGGAGGCTGCATTTTGG
**Jα7**	GGACTACAGCAACAACAGACTTAC
**Cα-P**	CAGAACCTGCTGTGTACCAG
**Vα8-PCR-F**	CAGACAGAAGGCCTGGTCAC
**Cα-PCR-R**	TGGCGTTGGTCTCTTTGAAG
**Dβ2-5'**	GTAGGCACCTGTGGGGAAGAAACT
**Jβ2-3'**	TGAGAGCTGTCTCCTACTATCGATT
**Insulin-F**	CCACCCAGGCTTTTGTCAA
**Insulin-R**	ATGCTGGTGCAGCACTGATC

Supplementary references

1 Muaddi, H. *et al.* Phosphorylation of eIF2alpha at serine 51 is an important determinant of cell survival and adaptation to glucose deficiency. *Molecular biology of the cell* **21**, 3220-3231, doi:10.1091/mbc.E10-01-0023 (2010).

2 Ito, T., Yang, M. & May, W. S. RAX, a cellular activator for double-stranded RNA-dependent protein kinase during stress signaling. *The Journal of biological chemistry* **274**, 15427-15432 (1999).

3 Verfaillie, T. *et al.* PERK is required at the ER-mitochondrial contact sites to convey apoptosis after ROS-based ER stress. *Cell death and differentiation* **19**, 1880-1891, doi:10.1038/cdd.2012.74 (2012).

4 Han, A. P. *et al.* Heme-regulated eIF2alpha kinase (HRI) is required for translational regulation and survival of erythroid precursors in iron deficiency. *The EMBO journal* **20**, 6909-6918, doi:10.1093/emboj/20.23.6909 (2001).

5 Good-Jacobson, K. L. *et al.* PD-1 regulates germinal center B cell survival and the formation and affinity of long-lived plasma cells. *Nat Immunol.* **11**, 535-542 (2010).

6 Kometani, K. *et al.* Repression of the transcription factor Bach2 contributes to predisposition of IgG1 memory B cells toward plasma cell differentiation. *Immunity* **39**, 136-147 (2013).
